# Draft Genome Sequence of *Mycobacterium tuberculosis* KT-0204, Isolated in South Korea

**DOI:** 10.1128/genomeA.01519-15

**Published:** 2016-02-04

**Authors:** Mi-ran Yun, Seung Jung Han, Won Gi Yoo, Taesoo Kwon, Sanghyun Lee, Jong Seok Lee, Dae-Won Kim

**Affiliations:** a Division of Biosafety Evaluation and Control, Korea National Institute of Health, Korea Centers for Disease Control and Prevention, Chungbuk, Republic of Korea; b School of Biological Sciences, Seoul National University, Seoul, Republic of Korea; c Department of Environmental Medical Biology, Chung-Ang University, Seoul, Republic of Korea; d Department of Microbiology and Institute for Immunology and Immunological Diseases, Brain Korea 21 Plus Project for the Medical Sciences, Yonsei University College of Medicine, Seoul, Republic of Korea; e International Tuberculosis Research Center, Masanhappo-gu, Changwon, Republic of Korea

## Abstract

Here, we describe the draft genome sequence of *Mycobacterium tuberculosis* KT-0204, non-Beijing family. This sequence will reveal genes related to the evolution and adaptation of *M. tuberculosis* KT-0204 in human hosts.

## GENOME ANNOUNCEMENT

Tuberculosis (TB) is one of the main infectious causes of death worldwide, with more than 9 million new cases of active disease diagnosed every year resulting in nearly 2 million deaths (World Health Organization, 2014) (1). Although Beijing family strains were assumed to be the predominant strains causing active TB in China, different sublineages of non-Beijing strains have been detected in the countries and regions adjacent to Mainland China (2–5). Thus, non-Beijing strains may also contribute to the high TB burden in Asia.

*Mycobacterium tuberculosis* KT-0204 is a member of the non-Beijing family MTB according to spoligotyping, and was found to be susceptible to first-line anti-TB drugs. *M. tuberculosis* KT-0204 was isolated from the sputum of a retreatment TB patient at Masan National Hospital (MNH) in South Korea. *M. tuberculosis* KT-0204 was grown in 7H9 broth (Difco Laboratories, Detroit, MI, USA) supplemented with 10% (vol/vol) oleic acid-albumin-dextrose-catalase (OADC; Becton Dickinson, Sparks, MD, USA) for 1 month at 37°C, and the genomic DNA was isolated.

A paired-end sequencing library was constructed with a 500-bp insert size using the NexTera sample preparation kit (Illumina, San Diego, CA, USA) for the Illumina-MiSeq platform. We produced a total of 5,088,454 reads from whole-genome sequencing with 311.14 fold coverage. We assembled the reads into 115 contigs with the CLC Genomics Workbench program (CLCbio, version 7.5) (6). The *N*_50_ size of the contigs was 102,547. After the assembly, we found that the KT-0204 strain has a genome of 4,377,418 bp with a 65.57% G+C content. From the assembled contigs, we identified 4,149 putative open reading frames (ORFs) with Glimmer (version 3.02) (7). To identify RNA genes, we used tRNAScan-SE (8) and RNAMMER (9), and 45 tRNAs and 3 rRNA genes were identified. From the 4,114 ORFs, we could assign clusters of orthologous group (COG) functional categories for 2,899 genes. The majority of genes were assigned to the COG functional categories cell wall/membrane/envelope biogenesis-related genes (126 genes, 4.35%) and lipid transport and metabolism-related genes (253 genes, 8.73%), except for general function genes (412 genes, 14.21%). *M. tuberculosis* has more than 100 outer membrane proteins and lipids required to construct the outer membrane (10), which explains why lipid transport and metabolism-related genes are particularly abundant in KT-0204. Moreover, we identified 1,936 single nucleotide variants (SNVs) and 176 indels against the *M. tuberculosis* H37Rv genome (accession number NC_000962) with GATK (11) (version 3.2.2). Among the identified SNVs, 78.7% (1,401) were in ORFs. For indels, 59.5% (58/118) of the insertions and 67.3% (37/58) of the deletions were in ORFs.

This genome will provide insight into the evolution and adaptation of *M. tuberculosis* KT-0204 in human hosts.

### Nucleotide sequence accession number.

This whole-genome shotgun project has been deposited in the DDBJ/EMBL/GenBank under the accession number JUFW00000000.
